# Dissociative symptomatology mediates the relation between posttraumatic stress disorder severity and alcohol‐related problems

**DOI:** 10.1111/acer.14764

**Published:** 2022-02-18

**Authors:** Herry Patel, Charlene O’Connor, Krysta Andrews, Michael Amlung, Ruth Lanius, Margaret C. McKinnon

**Affiliations:** ^1^ Department of Psychiatry and Behavioural Neurosciences McMaster University Hamilton Ontario Canada; ^2^ Homewood Health Care Guelph Ontario Canada; ^3^ Department of Applied Behavioral Science University of Kansas Lawrence Kansas USA; ^4^ Cofrin Logan Center for Addiction Research and Treatment University of Kansas Lawrence Kansas USA; ^5^ Department of Psychiatry Western University London Ontario Canada; ^6^ Homewood Research Institute Guelph Ontario Canada; ^7^ Mental Health and Addictions Program St Joseph’s Healthcare Hamilton Hamilton Ontario Canada

**Keywords:** alcohol, dissociation, posttraumatic stress disorder, treatment‐seeking

## Abstract

**Background:**

Up to 50% of individuals with posttraumatic stress disorder (PTSD) endorse problematic alcohol use. Typically, these individuals present with more complex and often more severe PTSD symptoms than those who do not report problematic alcohol use. Emerging literature suggests that heightened symptoms of dissociation are likewise associated with greater PTSD symptom severity. Despite this knowledge, the role of dissociation in the relation between PTSD severity and alcohol‐related problems has yet to be examined. Here, we explore the mediating role of dissociative symptomatology on the association between PTSD severity and alcohol‐related problems within a PTSD treatment‐seeking sample.

**Methods:**

Structural equation modeling was used to test the mediating role of dissociative symptomatology between PTSD severity and alcohol‐related problems. Participants [*N* = 334; mean age (SD) = 44.29 (9.77), 50% female] were drawn from a clinical intake battery database for PTSD in‐patient treatment services at Homewood Health Care, Guelph, ON, Canada. A subset of battery measures assessing PTSD severity, dissociative symptomatology, and alcohol‐related problems were submitted to analysis.

**Results:**

A significant positive association emerged between PTSD severity and alcohol‐related problems (*β* = 0.127, *p* < 0.05) in the absence of dissociative symptomatology. Critically, however, when added to this model, dissociative symptomatology (six unique facets of dissociation assessed by the Multiscale Dissociation Inventory) mediated the relation between PTSD severity and alcohol‐related problems. Specifically, greater PTSD severity was associated with greater dissociative symptomatology (*β* = 0.566, *p* < 0.0001), which was in turn associated with greater alcohol‐related problems (*β* = 0.184, *p* < 0.05).

**Conclusions:**

These results suggest that dissociative symptomatology plays a key role in explaining the relation between PTSD severity and alcohol‐related problems. Future studies should examine the impact of targeting dissociative symptomatology specifically in treating individuals with PTSD who endorse alcohol‐related problems.

## Graphical abstract

Posttraumatic Stress Disorder (PTSD) is frequently comorbid with alcohol use and its related problems. Dissociation is associated with greater severity of PTSD symptoms. However, the mediating role of dissociation on the relation between PTSD severity and alcohol related problems has not been examined. Our study illustrates that dissociation plays a key mediating role in the relation between PTSD severity and alcohol related problems. Thus, targeting dissociative symptomatology in treatment may prove helpful for individuals with PTSD endorsing problematic alcohol use.

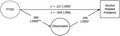

## INTRODUCTION

Posttraumatic stress disorder (PTSD) develops following exposure to a traumatic event (American Psychological Association, 2013) and is a highly prevalent and functionally de‐habilitating (Norman et al., 2007; Westphal et al., 2011) mental health disorder with 7%–8% of the population meeting criteria for PTSD at some point in their lives (Kessler et al., 1995). Here, symptoms of PTSD are categorized into four clusters: intrusive thoughts and memories, avoidance behaviors, Negative Alterations in Cognition and Mood (NACM), and alterations in arousal (American Psychological Association, 2013). Earlier work surrounding the self‐medication hypothesis posits that individuals with PTSD may use substances to cope with their symptoms (Khantzian, 1985). Indeed, PTSD is often associated with alcohol and/or substance use, with data from the 2010 National Epidemiologic Survey on Alcohol and Related Conditions (*N* = 34,653) estimating that 46.4% of individuals meeting criteria for PTSD also meet criteria for a substance use disorder (SUD; Pietrzak et al., 2011). Moreover, a 2016 follow‐up on this survey confirmed that these rates of alcohol and/or substance use remained stable over the intervening 6‐year period highlighting the high prevalence rate was not overestimated in the previous survey (Smith et al., 2016).

Among Canadians where the present study is situated, 9.6% of individuals meet criteria for PTSD with 27.8% of those individuals reporting alcohol use/dependence (Van Ameringen et al., 2008). Critically, comorbid PTSD and AUD/SUD are often associated with a heightened risk of other mental health concerns, suicidality, mortality, and functional impairment (Kilpatrick et al., 2003; Pietrzak et al., 2011), rendering it essential to examine the associations between PTSD and AUD/SUD to inform evidence‐based treatment methodologies.

A wide body of literature points to dissociation as a highly salient feature of PTSD, where the DSM‐5 including a dissociative subtype of PTSD that must be explored in any patient presenting for clinical assessment of the disorder (American Psychological Association, 2013). Here, dissociation is thought to involve detachment from immediate somatic or environmental experience, occurring during acute trauma and thus modulating its immediate psychophysiological impact (Spiegel, 2012). Put simply, dissociation is a psychological escape when no physical escape is possible (Putnam, 1991). Typically, individuals with the dissociative subtype of PTSD present with a history of more severe early‐life trauma (Stein et al., 2013) and higher PTSD severity scores (Wolf et al., 2012) than those without the subtype.

Notably, dissociation has also been linked to alcohol (Craparo et al., 2014; Evren et al., 2011; Zdankiewicz‐Ścigała & Ścigała, 2018) and/or substance use (Najavits & Walsh, 2012; Schäfer et al., 2010; Wenzel et al., 1996). Prior reports have examined dissociation as a predictor variable with alcohol or substance use and its related problems as outcomes (Craparo et al., 2014; Evren et al., 2011; Najavits & Walsh, 2012; Schäfer et al., 2010; Wenzel et al., 1996). After controlling for PTSD severity, the majority of these studies reveal significant associations between dissociative symptomatology and alcohol and/or substance use and its related problems even in the presence of PTSD severity (Evren et al., 2011; Najavits & Walsh, 2012), as well as with childhood adversity (Evren et al., 2011; Schäfer et al., 2010) and alexithymia (Craparo et al., 2014). Interestingly, a related study found associations between dissociative symptomatology and alcohol and/or cocaine use and its related problems for lifetime use but not recent use (Wenzel et al., 1996). Taken together, these studies point toward a clear association of PTSD severity and dissociation with both alcohol use and problems related to use. To note, PTSD psychopathology has been more closely linked to alcohol‐related problems rather than consumption (Angkaw et al., 2015; Bulloch et al., 2012; Simons et al., 2018; Wilson et al., 2017). Critically, however, despite this wide body of knowledge establishing key associations between PTSD and dissociation, PTSD and alcohol, and alcohol and dissociation, the potential unifying mechanisms underlying these three variables have yet to be explored.

In numerous previous reports, dissociation has been included as an independent variable of interest in relation to alcohol use and its related problems (Craparo et al., 2014; Evren et al., 2011; Najavits & Walsh, 2012; Schäfer et al., 2010; Wenzel et al., 1996). To our knowledge, however, no studies have examined dissociation as a mediator between PTSD severity and alcohol‐related problems. Accordingly, the primary objective of the present study was to examine the mediating effects of dissociative symptomatology on the well‐established relation between PTSD severity and alcohol‐related problems among a treatment‐seeking clinical sample meeting diagnosis for PTSD. Given the cross‐sectional nature of these data, an alternate model was also tested to examine PTSD severity as a mediator between dissociative symptomatology and alcohol‐related problems. Consistent with prior literature (Dworkin et al., 2018; McGlinchey et al., 2021; Patel et al., 2021; Walton et al., 2018), a secondary exploratory objective was to examine the mediating role of dissociation between specific PTSD symptom clusters and alcohol‐related problems.

## METHODS

### Participants

Participants were drawn from a clinical intake battery database from the Program for Traumatic Stress Recovery at Homewood Health Care, Guelph, ON. The program is a specialized in‐patient treatment program for individuals seeking treatment for PTSD. To be admitted to the program, individuals must: (1) be 18 years of age or older and (2) be able to participate in group therapy in a mixed community milieu.

Participants were informed their data would be used for research purposes; however, each individual was provided with the option to opt‐out from having their data be used for research with no penalty to their care. There was no compensation for participants as assessments were part of standard care. The research database was approved by the Homewood Research Ethics Board (REB #18‐07). A total of 402 clients completed the clinical intake battery between September 2017 and November 2019. Of these, nine participants were excluded due to readmissions to the program and 59 participants were excluded due to missing data on the dependent variable of interest. The final sample consisted of 334 individuals receiving inpatient care for PTSD as deemed necessary by a clinician and met the 33+ cut‐off score for provisional PTSD diagnosis on the PTSD Checklist (PCL‐5; Blevins et al., 2015).

### Measures

Data were collected occurred during the first week of a client's admission to the program. All measures were administered electronically to participants using Voxco Survey software. The battery included self‐report questionnaires assessing demographics, mental health screens, alcohol use, and psychological assessments. Demographic variables included age and sex, provided by participants at admission. Self‐report measures included validated assessments for psychiatric disorders and mechanisms/predictors of psychiatric disorder severity. A subset of these measures was analyzed in the present study, as described below.

#### PTSD severity

PTSD symptoms and severity in the past month were assessed using the PTSD Checklist for Diagnostic and Statistical Manual‐5 (PCL‐5; Blevins et al., 2015). The PCL‐5 is a 20‐item measure comprised of four clusters corresponding to the DSM‐5 diagnostic criteria: Cluster B (intrusive thoughts and memories), Cluster C (avoidance behaviors), Cluster D (NACM), and Cluster E (alterations in arousal). Scores on the PCL‐5 range from 0 to 80, with the following subscale ranges: Cluster B (0 to 20), Cluster C (0 to 8), Cluster D (0 to 28), and Cluster E (0 to 20). SEM was used to generate a single latent factor of PTSD symptoms from the cluster scores, as described below.

#### Dissociative symptomatology

The Multiscale Dissociation Inventory was used to assess dissociative symptoms within the past month (Briere et al., 2005). The MDI is a 30‐item self‐report questionnaire and measures six different types of dissociative response: (1) disengagement, (2) depersonalization, (3) derealization, (4) emotional constriction/numbing, (5) memory disturbance, and (6) identity dissociation. Items are rated on a scale of 1 (“never”) to 5 (“very often”). Scores on the MDI range from 30 to 150 and higher scores indicate greater dissociative symptomatology endorsement. SEM was used to generate a single latent factor of dissociative symptoms from the subscale scores, as described below.

#### Alcohol‐related problems

Alcohol use frequency and alcohol‐related problems experienced over the past 12 months were measured using the Alcohol Use Disorders Identification Test (AUDIT), a 10‐item self‐report screen assessing the frequency and severity of alcohol use (Saunders et al., 1993). The AUDIT assesses both consumption of alcohol (three items) and problems related to alcohol consumption (seven items). As such, typically within the literature, the AUDIT is referred to assessing alcohol‐related problems rather than consumption. Scores on the AUDIT range from 0 to 40 and a cut‐off of eight or greater indicates clinically hazardous alcohol use. The sum score on the AUDIT was included as the outcome variable in the mediation model.

#### Emotion dysregulation

The Difficulties in Emotion Regulation Scale (DERS) is a 36‐item self‐report questionnaire assessing six facets of emotion regulation within the past month: (1) non‐acceptance of emotional responses, (2) difficulty engaging in goal‐directed behavior, (3) impulse control difficulties, (4) lack of emotional awareness, (5) limited access to emotion regulation strategies, and (6) lack of emotional clarity (Gratz & Roemer, 2004; Hallion et al., 2018). Items are rated on a scale of 1 (“almost never”) to 5 (“almost always”). Scores on the DERS range from 36 to 180 and higher scores indicate more difficulty in emotion regulation. DERS sum score was utilized as a covariate in the mediation model.

#### Childhood adversity

The Adverse Childhood Experience Scale (ACES) is a 10‐item self‐report questionnaire used to assess the endorsement of adverse childhood experiences (Merrick et al., 2017). Participants are asked to indicate “yes” or “no” to a variety of adverse childhood experiences. Scores on the ACES range from 0 to 10. Higher scores indicate greater endorsement of adverse childhood experiences. A sum score on the ACES was used as a covariate in the mediation model.

### Data analysis plan

Descriptive statistics and zero‐order correlations were run among the study variables. Structural equation modeling was implemented in MPlus Version 8.4 (Muthén & Muthén, 1998‐2017) using maximum likelihood estimation to assess the mediating effects of dissociative symptomatology on the association between PTSD severity and alcohol‐related problems. The following latent factors were used within the mediation model. A latent factor for PTSD was created using the four symptom cluster scores from the PCL‐5: intrusions (Cluster B), avoidance (Cluster C), NACM (Cluster D), and alterations in arousal (Cluster E; refer to Figure S1 for measurement model). A latent factor for dissociative symptomatology was created using the six subscales of dissociation on the MDI: disengagement, depersonalization, derealization, emotional constriction/numbing, memory disturbance, and identity dissociation (refer to Figure S2 for measurement model). The mediation model tested the direct and indirect (mediated by dissociation) effects of PTSD severity on alcohol‐related problems. An alternate model was specified, using the same variables as above, in which the dissociation and PTSD latent factors were switched to account for the cross‐sectional nature of the data. An exploratory model for our secondary objective was also conducted examining the mediatory effects of dissociative symptomatology on the individual PTSD symptom clusters rather than global PTSD severity indicated by the latent factor. The secondary mediation model tested the direct and indirect (mediated by dissociation) effects of the four PTSD symptom clusters [intrusions (Cluster B), avoidance (Cluster C), NACM (Cluster D), and alterations in arousal (Cluster E)] on alcohol‐related problems while controlling for age, sex, emotion dysregulation, and childhood adversity. Before modeling, the alcohol‐related problems measure (AUDIT sum score) and the Identity Dissociation subscale from the MDI were square‐root transformed based on benchmarks of skewness >2 and kurtosis >7 (Curran et al., 1996). There were no significant outliers within the sample (*Z*s > 3.29). We used two‐tailed tests with a statistical significance of *α* < 0.05. Following established conventions (Hu & Bentler, 1999; Schreiber et al., 2006), an excellent‐fitting model has a comparative fit index (CFI) and Tucker Lewis index (TLI) ≥0.95, standardized root mean squared residual (SRMR) ≤0.08, and root mean square error of approximation (RMSEA) ≤0.06.

## RESULTS

### Descriptive statistics

The sample consisted of 334 participants (50% female) with a mean age of 44.29 (SD = 9.77). Sample characteristics can be found in Table 1. 26.6% of the sample met criteria for hazardous alcohol use (i.e., ≥8 on AUDIT). Furthermore, 88.6% of the sample reported at least one adverse childhood experience. Depression (*M* = 23.14) and anxiety (*M* = 21.18) scores were moderate within the sample (refer to Table 1) given the range of scores (0 to 42) on each measure.

**TABLE 1:** Sample characteristics

	Mean (standard deviation)
Age	44.29 (9.77)
Gender (%)	50% female
Education (%)	38.3% (diploma/bachelor's degree) 26.3% (some college/university)
Depression	23.14 (10.68)
Anxiety	21.18 (10.11)
Childhood Adverse Event Endorsement	88.6% (≥1 adverse event)
AUDIT total	6.19 (8.2)
Hazardous alcohol use (%)	26.6%
PCL Total	57.99 (11.06)
Avoidance	6.38 (1.70)
Intrusions	14.08 (3.79)
Negative Alterations	21.01 (4.30)
Reactivity	16.52 (4.11)
MDI Total	76.39 (22.60)
Disengagement	17.72 (4.23)
Depersonalization	10.90 (4.99)
Derealization	12.57 (4.78)
Emotional Constriction	14.69 (5.83)
Identity Dissociation	7.17 (3.74)
Memory Disturbances	13.33 (5.08)

Note

All values presented are mean (standard deviation) except for gender, education, childhood adversity endorsement, and hazardous alcohol use which are percentages.

### Primary mediation analysis

Prior to the mediation analyses, measurement models were specified for PTSD severity and dissociative symptomatology (as described prior). Measurement models for both latent variables can be found in the Supplemental Materials. Prior to the mediation analyses, the association between PTSD severity and alcohol‐related problems was first examined. The pathway from PTSD severity to alcohol‐related problems was examined with age and sex included as covariates within the model. The path from PTSD severity to alcohol‐related problems was significant (*β* = 0.127, *p* = 0.03). Among covariates, age was significantly associated with alcohol‐related problems (*β* = −0.198, *p* < 0.001). Sex, emotional dysregulation, and childhood adversity were not significantly associated with any of the variables and were trimmed from the model for parsimony.

**FIGURE 1:**
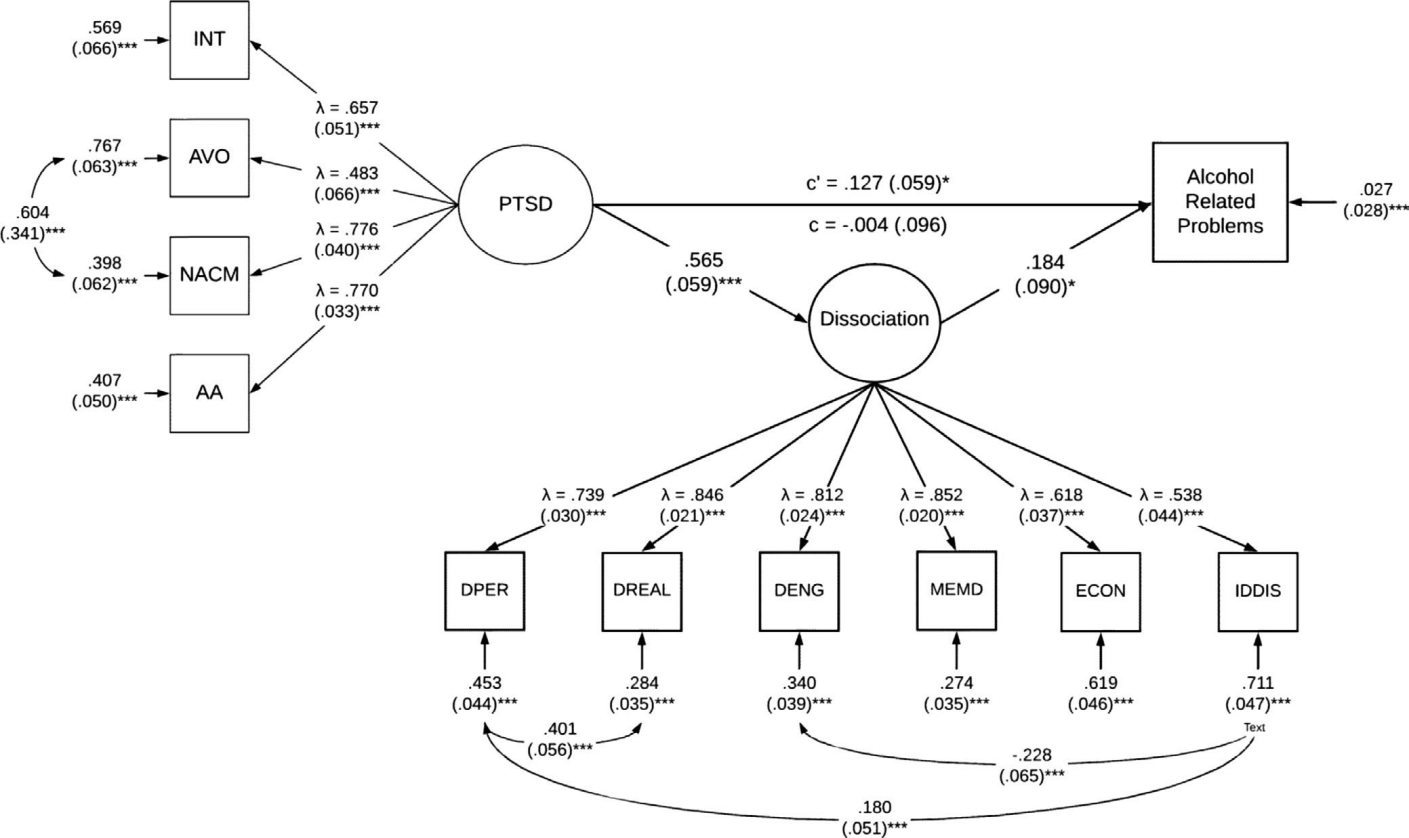
Primary mediation model assessing effects of dissociative symptomatology on the relation between PTSD severity and alcohol‐related problems. All values are standardized. Standard error for residuals and covariances in parentheses. PTSD = PTSD latent factor; INT = Intrusions; AVO = Avoidance; NACM = Negative Alterations in Cognition and Mood; AA = Alterations in Arousal; DPER = Depersonalization; DREAL = Derealization; DENG = Disengagement; MEMD = Memory Disturbance; ECON = Emotional Constriction/Numbing; IDDIS = Identity Dissociation. Age, sex, emotion dysregulation, and childhood adversity were controlled for. Model fit was adequate (X^2^(56, *N* = 334) = 125.67, *p* < 0.0001, TLI = 0.946, CFI = 0.961, RMSEA = 0.06 [95% CI = 0.047 to 0.075], SRMR = 0.04). *p < 0.05; **p < 0.01; ***p < 0.001

When evaluating the mediating effects of dissociative symptomatology, the paths between (1) PTSD severity and alcohol‐related problems, (2) PTSD severity and dissociative symptomatology, and (3) dissociative symptomatology and alcohol‐related problems were assessed. Covariates in the model include age, sex, emotional dysregulation, and childhood adversity. Among covariates, sex and childhood adversity were not significantly associated with any other variables and thus were trimmed from the model for parsimony. Age was significantly associated with alcohol‐related problems (*β* = −0.187, *p* < 0.001) but not dissociative symptomatology (*β* = −0.019, *p* = 0.690), whereas emotional dysregulation was significantly associated with dissociative symptomatology (*β* = 0.213, *p* < 0.001) but not alcohol‐related problems (*β* = 0.006, *p* = 0.933). In the presence of the significant covariates and dissociative symptomatology added as a mediator, the direct path from PTSD severity to alcohol‐related problems (*β* = −0.004, *p* = 0.971) was no longer significant. The path from PTSD severity to dissociative symptomatology was significant (*β* = 0.565, *p* < 0.001) and the path from dissociative symptomatology to alcohol‐related problems was significant (*β* = 0.184, *p* = 0.04). Refer to Figure 1 for the mediation model including direct and indirect effects of PTSD severity on alcohol‐related problems.

### Alternate mediation model

**FIGURE 2:**
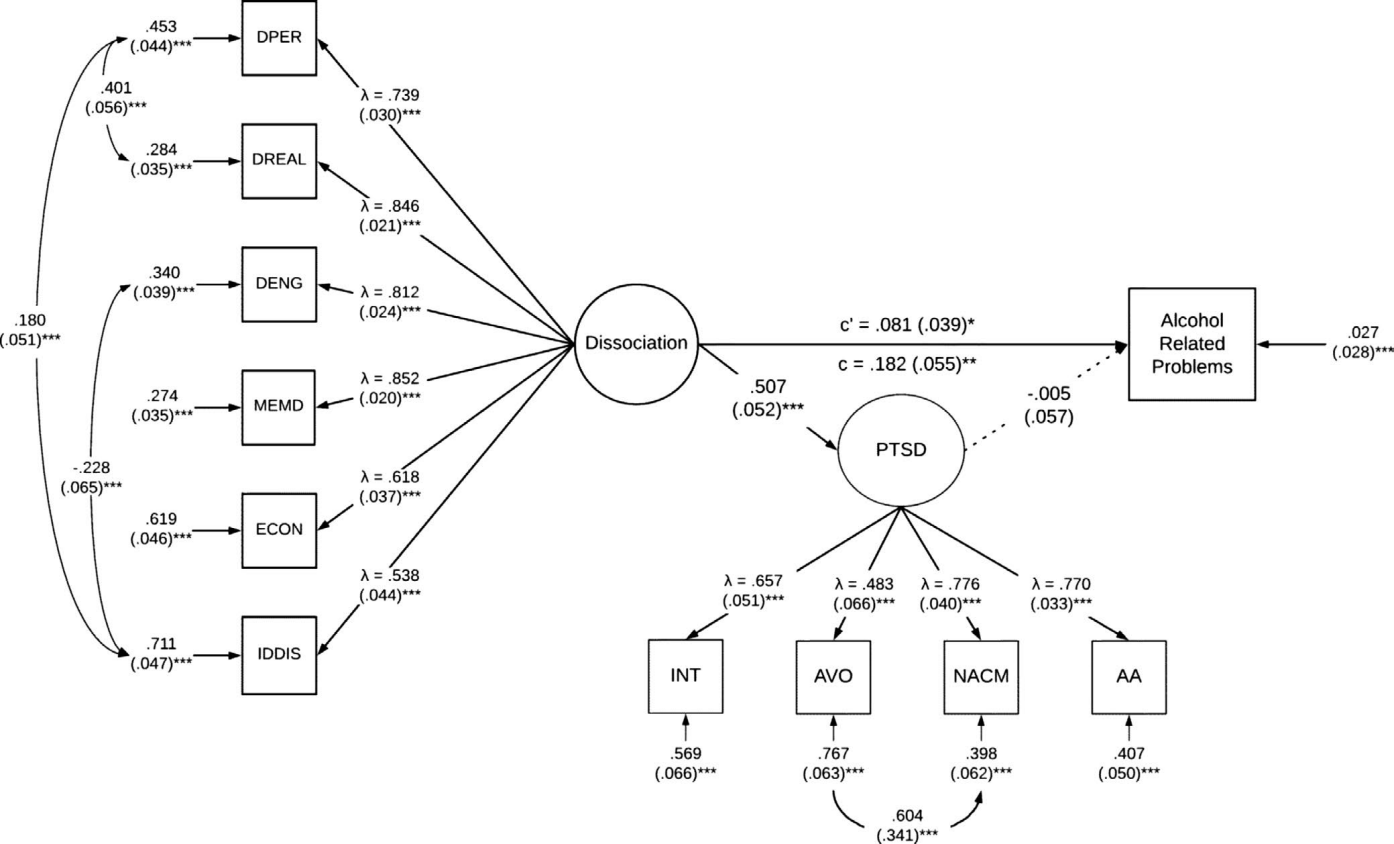
Alternate mediation model assessing effects of PTSD severity on the relation between dissociative symptomatology and alcohol‐related problems. All values are standardized. Standard error for residuals and covariances in parentheses. PTSD = PTSD latent factor, INT = Intrusions, AVO = Avoidance NACM = Negative Alterations in Cognition and Mood, AA = Alterations in Arousal, DPER = Depersonalization, DREAL = Derealization, DENG = Disengagement, MEMD = Memory Disturbance, ECON = Emotional Constriction/Numbing, IDDIS = Identity Dissociation. Age, sex, emotion dysregulation, and childhood adversity were controlled for. Model fit was adequate (X^2^(58, *N* = 334) = 150.54, *p* < 0.0001, TLI = 0.931, CFI = 0.948, RMSEA = 0.07 [95% CI = 0.056 to 0.083], SRMR = 0.05). *p < 0.05; **p < 0.01; ***p < 0.001

To account for the cross‐sectional nature of the data, an alternate model was specified using the same variables as the primary mediation model; however, the predictor and mediator variables were switched. Here, PTSD severity was specified as the mediator and dissociative symptomatology was specified as the predictor. Thus, this alternate model tested the paths from (1) dissociative symptomatology to alcohol‐related problems, (2) dissociative symptomatology to PTSD severity, and (3) PTSD severity to alcohol‐related problems. Age was the only significant covariate in the model given a significant association with alcohol‐related problems. Sex, emotion dysregulation, and childhood adversity were not significantly associated with anything and thus were subsequently trimmed from the model for parsimony. The direct path from dissociative symptomatology to alcohol‐related problems (*β* = 0.081, *p* < 0.05) was still significant in the presence of PTSD severity as a mediator. The path from dissociative symptomatology to PTSD severity was significant (*β* = 0.507, *p* < 0.001); however, the path from PTSD severity to alcohol‐related problems was not significant (*β* = −0.005, *p* > 0.05). Refer to Figure 2 for the alternate mediation model including direct and indirect effects of dissociative symptomatology on alcohol‐related problems.

### Secondary cluster model

A secondary model was specified in which the PTSD latent factor was replaced with the individual symptom clusters: (1) intrusions (Cluster B), avoidance (Cluster C), NACM (Cluster D), and alterations in arousal (Cluster E) to examine direct and indirect (mediated by dissociation) associations between specific symptoms clusters and alcohol‐related problems. Similar to the primary model, before assessing the mediating effects of dissociation, pathways between each symptom cluster and alcohol‐related problems were assessed with age, sex, emotion dysregulation, and childhood adversity as covariates. Only the alterations in arousal cluster was significantly associated with alcohol‐related problems (*β* = −0.191, *p* < 0.001); the other symptom clusters were not significantly associated with alcohol‐related problems (intrusions: *β* = −0.039, *p* = 0.554; avoidance: *β* = 0.015, *p* = 0.799; NACM: *β* = −0.046, *p* = 0.534). Among covariates, only age was significantly associated with alcohol‐related problems; sex, emotion dysregulation, and childhood adversity were not significantly associated with alcohol‐related problems and were subsequently trimmed from the model for parsimony.

**FIGURE 3:**
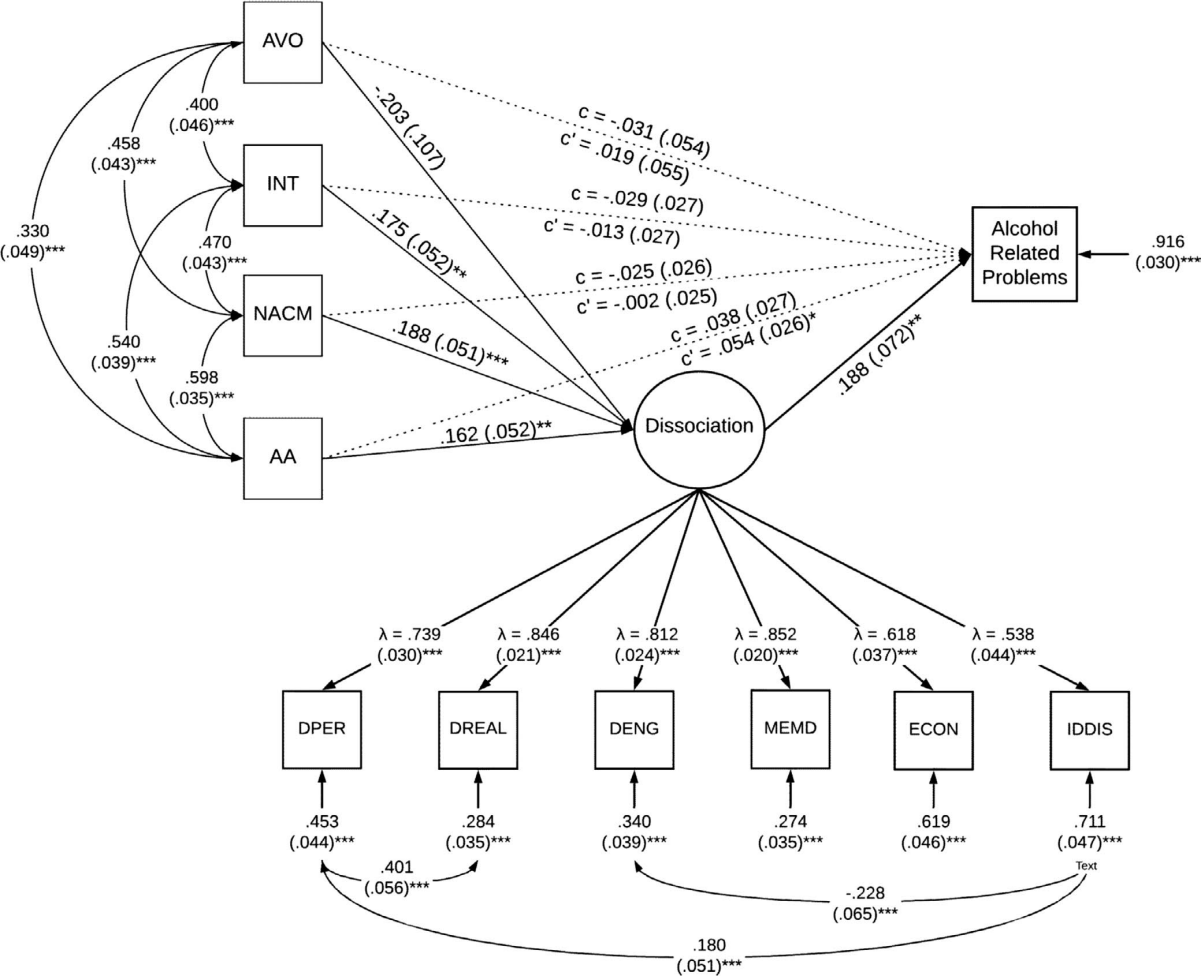
Secondary mediation model assessing effects of dissociative symptomatology on the relation between PTSD symptom clusters and alcohol‐related problems. All values are standardized. Standard error for residuals and covariances in parentheses. PTSD = PTSD latent factor, INT = Intrusions, AVO = Avoidance NACM = Negative Alterations in Cognition and Mood, AA = Alterations in Arousal, DPER = Depersonalization, DREAL = Derealization, DENG = Disengagement, MEMD = Memory Disturbance, ECON = Emotional Constriction/Numbing, IDDIS = Identity Dissociation. Age, sex, emotion dysregulation, and childhood adversity were controlled for. Model fit was adequate (X^2^(43, *N* = 334) = 89.42, *p* < 0.0001, TLI = 0.948, CFI = 0.965, RMSEA = 0.06 [95% CI = 0.040 to 0.073], SRMR = 0.03). *p < 0.05; **p < 0.01; ***p < 0.001

When evaluating the mediating effects of dissociative symptomatology, the paths between: (1) each PTSD symptom cluster and alcohol‐related problems, (2) each symptom cluster and dissociative symptomatology, and (3) dissociative symptomatology and alcohol‐related problems were assessed. Covariates in the model include age, sex, emotional dysregulation, and childhood adversity. Sex and childhood adversity were not significantly associated with any other variables and thus were trimmed from the model for parsimony. Whereas age was significantly associated with alcohol‐related problems (*β* = −0.185, *p* = 0.001) but not dissociative symptoms (*β* = 0.014, *p* = 0.772), emotion dysregulation was associated with dissociative symptomatology (*β* = 0.321, *p* < 0.001) but not alcohol‐related problems (*β* = 0.008, *p* = 0.912). All direct paths from each PTSD symptom cluster and alcohol‐related problems were no longer significant (intrusions: *β* = −0.07, *p* = 0.296; avoidance: *β* = 0.032, *p* = 0.599; NACM: *β* = −0.07, *p* = 0.346; alterations in arousal: *β* = 0.100, *p* = 0.166). Paths from each PTSD symptom cluster to dissociative symptomatology were significant for: intrusions (*β* = 0.189, *p* = 0.001), NACM (*β* = 0.229, *p* < 0.001), and alterations in arousal (*β* = 0.189, *p* = 0.002) but not avoidance (*β* = −0.098, *p* = 0.058). The path from dissociative symptomatology to alcohol‐related problems was significant (*β* = 0.192, *p* = 0.008). Please refer to Figure 3 for mediation model including direct and indirect effects of each PTSD symptom cluster on alcohol‐related problems.

## DISCUSSION

To our knowledge, this was the first study to examine the mediatory effects of dissociative symptomatology on the relation between PTSD severity and alcohol‐related problems among a treatment‐seeking sample of patients diagnosed with PTSD. The results are consistent with the hypothesis that dissociation significantly and positively mediates the relation between PTSD severity and alcohol‐related problems. Greater PTSD severity was associated with heightened dissociative symptoms, which in turn was associated with greater alcohol‐related problems. This pattern of results was not observed in the alternate model, where greater dissociative symptoms were associated with heightened PTSD severity, but greater PTSD severity did not significantly associate with alcohol‐related problems. The lack of significant mediating effect by PTSD severity indicates a unique mediating role of dissociative symptomatology between PTSD severity and alcohol‐related problems. Notably, exploratory analyses examining the individual symptom clusters of PTSD further support the mediating role of dissociative symptoms between intrusions, NACM, and alterations in arousal with alcohol‐related problems; the same mediating effects were not observed for the avoidance symptom cluster.

Interestingly, there was a lack of significant sex differences seen in the current study which is inconsistent with prior findings in the literature with females showing greater severity of PTSD symptoms as compared to males (Ramikie & Ressler, 2018). Furthermore, males show greater prevalence of AUD as compared to females (Agabio et al., 2016); however, females show greater health‐related problems related to alcohol consumption than males (Agabio et al., 2016). Given that the sample was 50% female, it is interesting that sex and/or gender differences were not observed given significant power for analyses. A potential explanation for these inconsistent results includes an absence of gender assessment beyond the binary options of male and female within our sample, thus any gender‐related effects may have been eroded as patients were forced into a binary response for gender and sex. However, it is important to note that there is also a gender bias in seeking mental health care where women are more likely to seek help than men (Klose & Jacobi, 2004; Pattyn et al., 2015; Rhodes & Goering, 1994) thus gender bias is a limitation to be considered among established gender differences in the literature.

Dissociation has been linked previously to alcohol and substance use and its related problems (Wenzel et al., 1996), even after controlling for PTSD severity (Evren et al., 2011; Najavits & Walsh, 2012), childhood adversity (Evren et al., 2011; Schäfer et al., 2010), and alexithymia (Craparo et al., 2014). These prior findings are consistent with current results supporting a significant positive association between dissociation and alcohol‐related problems. The present study, however, was the first to examine the underlying mechanisms of this relation by revealing the mediating role of dissociative symptoms between PTSD severity and alcohol‐related problems. In addition to revealing this potential mechanism, these findings suggest that individuals with PTSD and heightened symptoms of dissociation are more likely to experience greater severity of alcohol‐related problems in comparison to individuals with PTSD not experiencing dissociation.

Taken together, the results of the current study are consistent with the self‐medication hypothesis, which suggests that individuals use substances and/or alcohol to cope with psychiatric symptoms (Khantzian, 1985). Given that the presence of heightened dissociation among individuals with PTSD appears related to greater functional impairment (Boyd et al., 2018), it is probable that those individuals with PTSD experiencing heightened levels of dissociation may also be more likely to use alcohol and/or substances maladaptively, thus heightening the risk of alcohol and/or substance‐related problems in this disorder. The specific targeting of dissociative symptoms may prove helpful as a clinical intervention for treatment‐seeking individuals with PTSD who also endorse problematic alcohol use. Indeed, there is a promising adjunctive treatment program for dissociative patients called the Treatment of Patients with Dissociative Disorders that helps stabilize emotion dysregulation and safety concerns among highly dissociated individuals (Brand et al., 2019).

The exploratory model examining the mediating role of dissociation between PTSD symptom clusters and alcohol‐related problems follows prior literature closely (Dworkin et al., 2018; McGlinchey et al., 2021; Patel et al., 2021; Walton et al., 2018) which has pointed toward significant relations between the intrusion, NACM, and alterations in arousal clusters of PTSD and alcohol misuse (Walton et al., 2018) in the absence of dissociation. In the present study involving a large number of treatment‐seeking inpatients, however, only the alterations in arousal symptom cluster associated directly with alcohol‐related problems in the absence of dissociation. By contrast, the intrusion and NACM clusters were significantly associated with alcohol‐related problems within this sample, but indirectly through dissociative symptoms, whereas alterations in arousal remained significantly associated with alcohol‐related problems indirectly through dissociation. To note, whereas Walton et al. (2018) found direct associations between alcohol misuse and each of the four PTSD symptom clusters among predominantly male, military veterans, to increase power, our sample comprised a heterogeneous group of multiple groups (military and civilians) experiencing PTSD. Moreover, 50% of the present sample identified as being of the female sex.

Interestingly, Dworkin et al. (2018) found differential PTSD cluster associations with alcohol and other SUDs. Whereas among individuals with alcohol use disorder, significant associations emerged between avoidance symptoms in the absence of other substance use, and among individuals with comorbid alcohol and cocaine use disorder, alterations in arousal were significantly associated with heightened PTSD symptom severity. Although the present study is somewhat inconsistent in failing to reveal an association between avoidance and alcohol‐related problems, we did observe a significant association between alcohol‐related problems and alterations in arousal, in the absence of dissociation. Indeed, when dissociation was included, alterations in arousal remained indirectly and significantly associated with alcohol‐related problems; avoidance problems, however, were not significantly associated suggesting dissociation may be a highly specific underlying mechanism of the relation between avoidance symptoms in PTSD and heightened alcohol abuse.

In a pattern inconsistent with our findings, Patel et al. (2021) did not find any significant associations between PTSD symptom clusters and alcohol‐related problems among a sample of individuals seeking treatment for concurrent disorders. Notably, the sample in Patel et al. (2021) was recruited from an outpatient clinic, where patients with primary alcohol use disorders were not treated, thus potentially accounting for the lack of any significant associations in this sample. Finally, McGlinchey et al. (2021) conducted a network analysis between symptoms of PTSD and alcohol use disorder among a sample of Irish military veterans and found the “reckless behavior” symptom (part of alterations in arousal cluster) yielded the strongest associations with alcohol use disorder symptoms. This finding, in particular, is consistent with the current findings linking alterations in arousal with alcohol‐related problems directly (in the absence of dissociation) and indirectly (through dissociative symptoms), thus lending further support to the hypothesis that alterations in arousal symptom cluster exert a critical role in the influence of PTSD severity on alcohol‐related problems. It is important to note that, despite the clinical utility of examining the relations between these variables, the model explored here was exploratory, thus warranting further urgently needed investigation.

Overall, the results of the present study align with the present literature and add to the field by identifying a potential mechanism of the association between PTSD and alcohol‐related problems. These findings are also clinically relevant given that PTSD and alcohol use are highly comorbid and alcohol use among individuals with PTSD has been linked to greater PTSD severity and reduced treatment response (Kilpatrick et al., 2003; Pietrzak et al., 2011). Moreover, dissociation has also been linked to alcohol use and its related problems (Craparo et al., 2014; Evren et al., 2011; Zdankiewicz‐Ścigała & Ścigała, 2018). Taken together, dissociation plays a unique role in the relation between PTSD severity and alcohol‐related problems making it a critical target for intervention among clinical samples to potentially increase treatment response among individuals with PTSD endorsing alcohol use.

This study is not without limitations. First, all data within the study were self‐report; all measures utilized within the study, however, are validated measures commonly used within the literature. Importantly, the AUDIT questionnaire is intended to be a screener for the potential presence of an AUD rather than assessing the severity of AUD symptoms, which is only done by a semi‐structured interview with a licensed clinician. Second, the term mediation is used throughout to describe the relation between PTSD severity, dissociation, and alcohol‐related problems; however, given these data are cross‐sectional, longitudinal studies are needed to determine the true mediating role of dissociative symptoms on the relation between PTSD severity and alcohol‐related problems. Third, another limitation is that the time frames for each of the self‐report measures are different given that the AUDIT assesses alcohol‐related problems within the past 12 months, whereas the PCL‐5 and MDI assess symptoms experienced within the past month. Fourth, all participants included within the sample met cut‐off scores for probable PTSD (≥33 according to the PCL‐5; Blevins et al., 2015). Thus, additional research will be required to further determine whether the present findings hold among trauma‐exposed but resilient individuals (subthreshold experiences of PTSD symptoms) who endorse alcohol use and its related problems.

In conclusion, this is the first study to reveal the mediating role of dissociation in the relation between PTSD severity and alcohol‐related problems. Future studies should not only examine the mediating role of dissociation among trauma‐exposed resilient individuals but also use longitudinal study designs to examine whether peri‐traumatic or posttraumatic dissociation emerges as the most significant mediator between PTSD severity and alcohol‐related problems. Furthermore, future studies should also evaluate gender differences in the mediating role of dissociation in the relation between PTSD symptom severity and alcohol‐related problems as it was not observed within this sample. On balance, the present findings point toward the critical need to explore clinically dissociative symptomatology among treatment‐seeking individuals with PTSD who endorse using alcohol. Identifying and subsequently targeting dissociative symptomatology clinically have the potential to enhance treatment efficacy among individuals who present with more complex clinical presentations of PTSD due to their comorbid alcohol use.

## CONFLICT OF INTEREST

The authors have no conflicts of interest to disclose.

## Supporting information

Supinfo S1
