# Supplementary material for: Dissociative symptomatology mediates the relation between posttraumatic stress disorder severity and alcohol‐related problems
Source: Alcohol Clin Exp Res. 2022 Feb 18;46(2):289–99. doi: 10.1111/acer.14764 (PMC9307004; doi:10.1111/acer.14764)

**Supplemental Materials**

**Figure S1. Latent PTSD Factor Measurement Model**

All values are standardized. Standard error for residuals and covariances in parentheses. PTSD = PTSD latent factor, INT = Intrusions, AVO = Avoidance NACM = Negative Alterations in Cognition and Mood, AA = Alterations in Arousal. Model fit was adequate (*χ* ^2^(1, *N* = 334) = 10.57, *p* = .01, TLI = .845, CFI = .974, RMSEA = .169 [95% CI = .088, .268], SRMR = 0.03). * *p* < .05; ** *p* < .01; *** *p* < .001


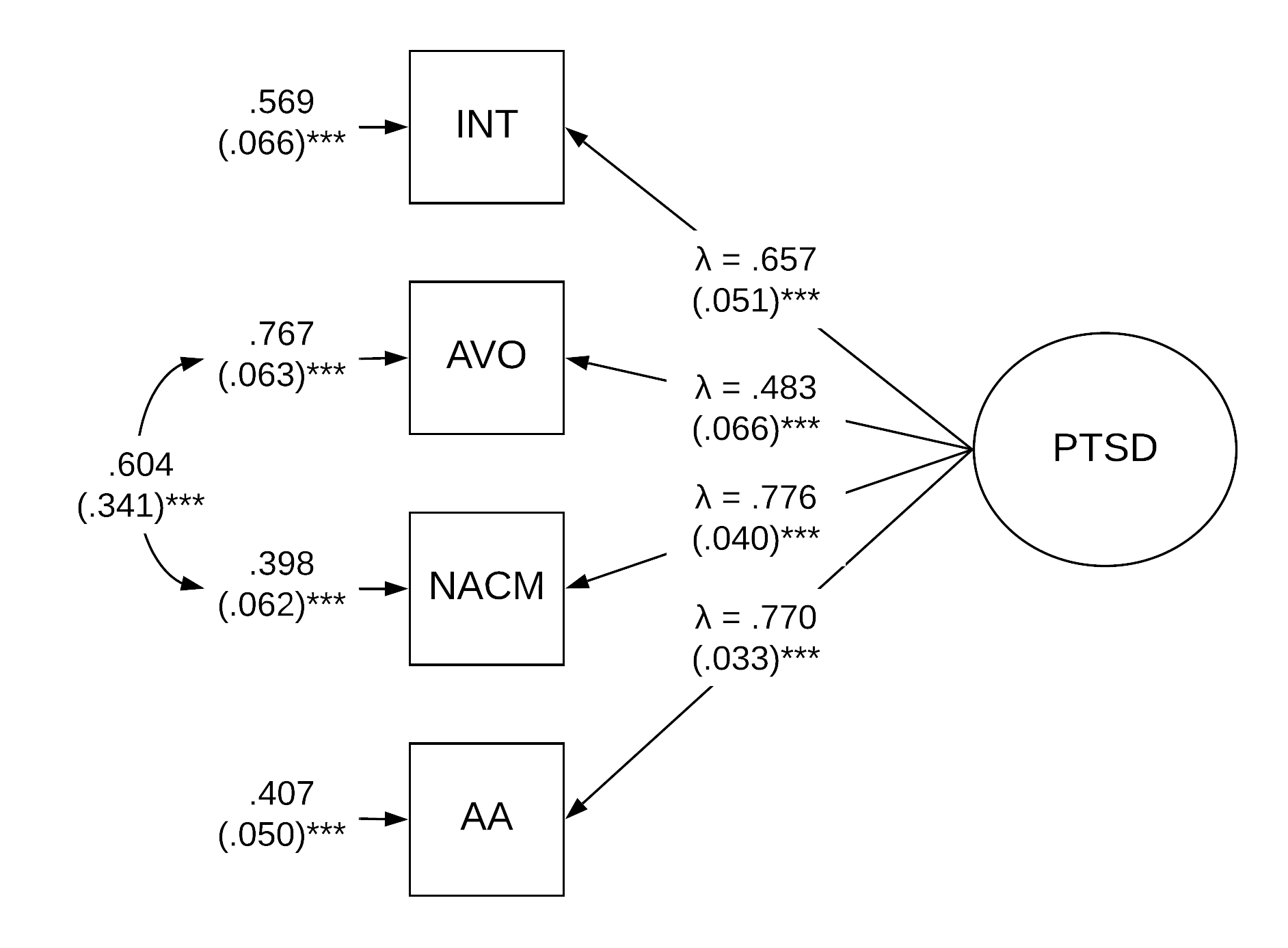


**Figure S2. Latent Dissociation Measurement Model.**

All values are standardized. Standard error for residuals and covariances in parentheses. DPER = Depersonalization, DREAL = Derealization, DENG = Disengagement, MEMD = Memory Disturbance, ECON = Emotional Constriction/Numbing, IDDIS = Identity Dissociation. Model fit was excellent (*χ* ^2^(6, *N* = 334) = 7.72, *p* > .05, TLI = .996, CFI = .998, RMSEA = 0.03 [95% CI = .000, .081], SRMR = 0.01). * *p* < .05; ** *p* < .01; *** *p* < .001


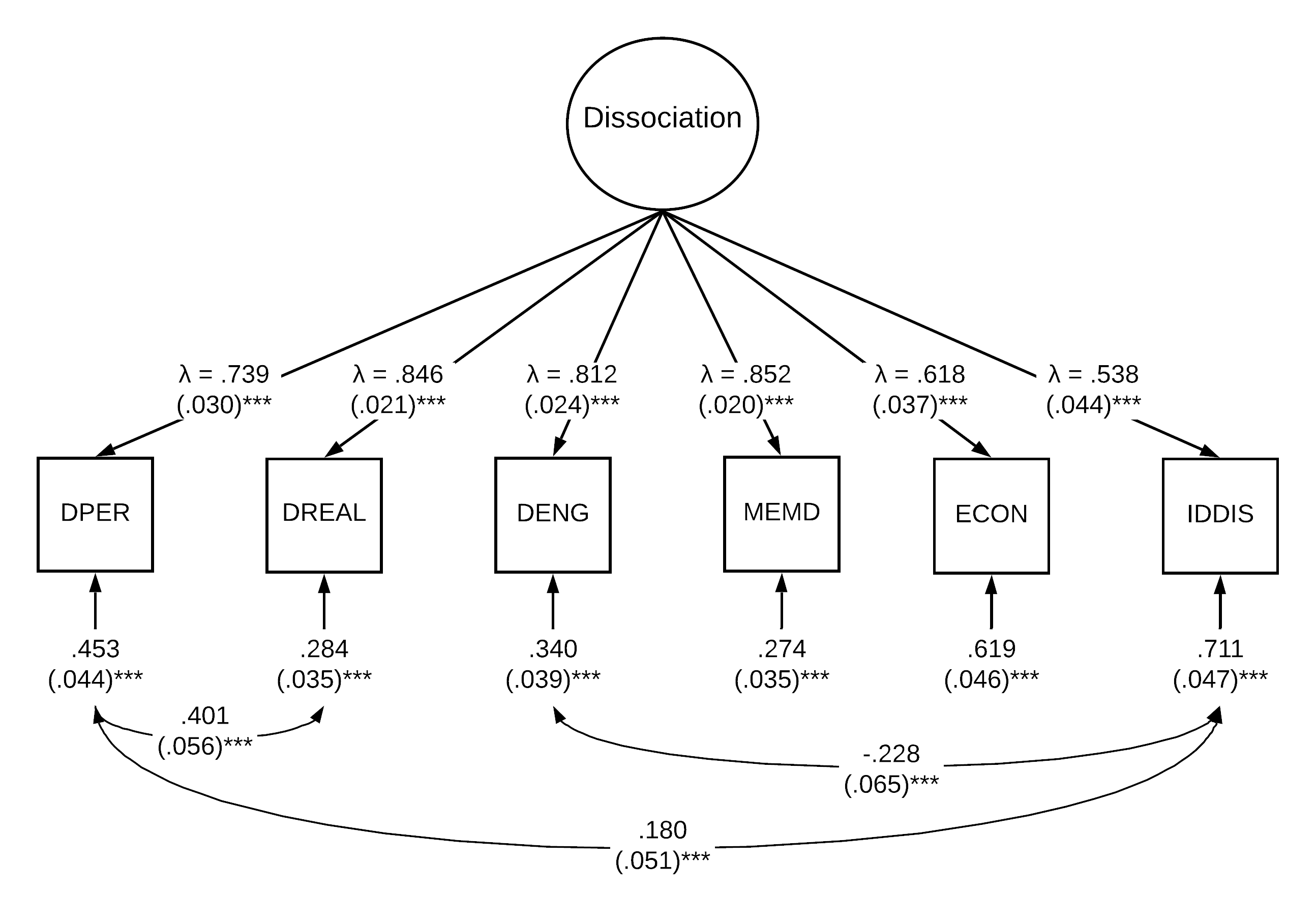

Supplement: Supplementary file 1 — Supinfo S1 [file ACER-46-289-s001.docx]
